# Robotic versus laparoscopic surgery: a comparative assessment of outcomes, complications, recovery, and cost

**DOI:** 10.25122/jml-2025-0139

**Published:** 2025-12

**Authors:** Ahmed Abdulrahman Al-Saeed, Ghannam Alghannam

**Affiliations:** 1 College of Medicine, King Faisal University, Al Hofuf, Saudi Arabia; 2 Poly Clinic, King Faisal University, Al Hofuf, Saudi Arabia

**Keywords:** robotic surgery, robot-assisted surgery, laparoscopic surgery, minimally invasive surgery, outcomes, complications, recovery time, cost, MIS, Minimally invasive surgery, RARP, Robotic-assisted radical prostatectomy, LRP, Laparoscopic Radical Prostatectomy, R-ISR, Robotic intersphincteric resection, L-ISR, Laparoscopic intersphincteric resection

## Abstract

Minimally invasive surgery (MIS) has revolutionized modern surgical practice, reducing trauma, speeding recovery, and improving surgical accuracy. Robot-assisted surgery has emerged as a minimally invasive surgical technique as an alternative to laparoscopic surgery. However, determining which of the two surgical techniques is more effective across different surgical specialties remains a matter of research. This narrative review aimed to compare robotic-assisted surgery with laparoscopic surgery with respect to patient outcomes, complications, recovery time, and cost. Studies published in PubMed and ScienceDirect were used, with a focus on studies published between 2015 and 2025. Also, studies that compared both techniques through various specialties were included. Robot-assisted surgery showed positive results by improving surgical precision, reducing the need for conversion to open surgery, and resulting in fewer complications and faster functional recovery in some procedures. However, laparoscopic surgery showed superior results in lowering surgical costs and ease of access, with shorter operating times than robotic-assisted surgery. Both robotic-assisted surgery and laparoscopic surgery offer advantages that make comparing them difficult. Robotic-assisted surgery was better for complex surgical procedures that required high precision. However, laparoscopic surgery was superior for routine cases requiring shorter operating times. Further research and prospective, high-quality, and multicenter studies are still needed to better define the optimal application of each surgical approach.

## INTRODUCTION

Minimally invasive surgery (MIS) is increasingly used in surgical practice, contributing to reduced trauma, faster recovery, and enhanced precision during surgical procedures. Robot-assisted surgery is emerging as an alternative to laparoscopy, offering multiple advantages such as three-dimensional imaging and improved dexterity [1]. However, determining which of the two techniques is more effective in terms of clinical outcomes remains under debate.

Several studies have comprehensively compared the effectiveness of robotic-assisted surgery with laparoscopic surgery across various surgical specialties. Many of these reviews found that robotic-assisted surgery was generally associated with lower complication rates and faster postoperative recovery [2,3]. However, laparoscopic surgery demonstrates significant advantages in terms of surgical time and cost-effectiveness [1,2].

In colorectal surgery, robotic-assisted surgery is associated with longer operative times but shorter hospital stays and fewer conversions to open surgery than laparoscopic surgery [2].

This narrative review aimed to compare robotic-assisted surgery with laparoscopic surgery in terms of patient outcomes, complication rates, recovery time, and cost. Although the number of specialized studies in this field is increasing, most focus on a specific surgical specialty or procedure to compare the two techniques, rather than providing a comprehensive comparison across different surgical specialties in a single study. This narrative review aims to fill this gap by analyzing and reviewing studies conducted between 2015 and 2025 and providing a comprehensive comparison of the two surgical techniques.

## MATERIAL AND METHODS

### Eligibility criteria

This narrative review aimed to compare robotic and laparoscopic surgery with respect to patient outcomes, complications, recovery time, and cost. A comprehensive literature search was conducted using PubMed and ScienceDirect, covering studies published between 2015 and 2025. The keywords used were: ‘robotic surgery’, ‘robot-assisted surgery’, ‘laparoscopic surgery’, ‘minimally invasive surgery’, ‘outcomes’, ‘complications’, ‘recovery time’, and ‘cost’.

Studies were included if they were published in English, provided a direct comparison between robotic-assisted surgery and laparoscopic surgery, focused on any surgical specialty, and reported at least one of the following outcomes: patient outcomes, complication rates, recovery time, or cost. Studies were excluded if they did not compare both surgical techniques or if they lacked sufficient outcome data.

### Search strategy

A comprehensive electronic search strategy was applied across all databases. In PubMed and ScienceDirect, the following Boolean search string was used: (‘robotic surgery’ OR ‘robot-assisted surgery’) AND ‘laparoscopic surgery’ AND (‘minimally invasive surgery’ OR ‘surgical outcomes’ OR ‘complications’ OR ‘recovery time’ OR ‘cost’). Search filters were set to include only articles published in English between 2015 and 2025. The most recent search was conducted on May 8, 2025.

### Study selection process

The selection process involved an initial screening of titles and abstracts to assess relevance. Full-text articles of potentially eligible studies were then reviewed according to the inclusion and exclusion criteria. Duplicate studies were removed. Due to the narrative nature of this review, no formal risk of bias assessment was performed.

### Outcomes

Patient outcomes were a key criterion for assessing the effectiveness of robotic surgery compared to laparoscopic surgery. These outcomes encompass several indicators, most notably patient safety during the surgical procedure, postoperative recovery, complication rates, and long-term functional recovery.

## RESULTS

Numerous studies examined the outcomes of colorectal surgery. A meta-analysis of 50,771 patients across 21 studies showed that robotic-assisted surgery (RAS) was associated with a longer time to perform the procedure and a shorter hospital stay than laparoscopic surgery (LS) [2]. As for secondary outcomes, laparoscopic surgery was associated with shorter operating time, which means it may be a faster option than robotic surgery for some procedures [3]. On the other hand, robotic-assisted surgery required fewer conversions to open surgery than laparoscopic surgery, indicating greater precision and a lower need for more invasive interventions [3].

Several studies compared robotic-assisted radical prostatectomy (RARP) with laparoscopic radical prostatectomy (LRP) in terms of clinical outcomes and health-related quality of life. A large meta-analysis of 46 studies, including four randomized controlled trials and 42 non-randomized trials, showed that robotic-assisted radical prostatectomy and laparoscopic radical prostatectomy were similar in several parameters, including blood loss, catheter intubation time, overall complication rate, and positive surgical margin rate [4]. However, non-randomized trials demonstrated advantages of robotic surgery, including less blood loss, shorter catheter intubation time, shorter hospital stay, lower blood transfusion rate, and reduced overall complication rate [4]. Furthermore, a meta-analysis of randomized trials demonstrated that robotic surgery was significantly superior to laparoscopic surgery in restoring urinary control and erectile function in the postoperative period [4]. Significant improvements in urinary control and erectile function were observed at all time points examined (1 to 12 months post-surgery) [4]. Regarding quality of life, a separate systematic review analyzed 23 studies that used validated assessment tools, such as the EORTC QLQ-C30, and found no significant long-term differences between RARP and LRP in general health status one year after surgery [5]. The RARP score was 76.3, compared with 76.1 for LRP [5]. However, RARP scores were associated with faster recovery and earlier return of urinary tract function, which may improve quality of life in the short term [5].

In a study comparing robotic-assisted benign hysterectomy with laparoscopic benign hysterectomy, patients’ outcomes were analyzed for blood loss, length of hospital stay, blood transfusion, conversion rate, and complications associated with each surgical technique [6]. The results showed that robotic-assisted benign hysterectomy was associated with 52.31 mL less blood loss than laparoscopic surgery, a reduced need for blood transfusions, and a shorter hospital stay [6]. The analysis also showed that robotic surgery was associated with a lower probability of postoperative hospital stays exceeding two days compared with laparoscopic surgery [6].

A study comparing robotic cholecystectomy and laparoscopic cholecystectomy showed that the robotic group had lower postoperative complication rates (3.8%) than the laparoscopic group (20.4%) [7]. Furthermore, the robotic surgery group experienced no procedure-related complications, whereas 3.5% were recorded in the laparoscopic group [7]. As for conversion to open surgery, no conversions were recorded in robotic surgery, while 1.9% of patients were converted in laparoscopic surgery [7]. The results also indicated that patients who underwent laparoscopic cholecystectomy required pain medications for a longer duration than patients in the robotic group [7]. Regarding the duration of the procedure, the results showed that robotic cholecystectomy took longer than laparoscopic cholecystectomy, at 75.7 minutes compared to 64.37 minutes [7]. Additionally, a retrospective study compared the outcomes of robotic cholecystectomy with laparoscopic cholecystectomy. Statistical matching was performed between the two groups —those undergoing robotic cholecystectomy and those undergoing laparoscopic cholecystectomy —to ensure balanced clinical characteristics [8]. The results showed that robotic surgery was associated with longer operative time compared to laparoscopic surgery [8]. On the other hand, robotic surgery was featured with shorter hospital stay and lower readmission rates within 90 days of surgery [8]. Despite all these advantages, robotic surgery was associated with higher costs and greater resource consumption [8].

Also, in a recent study, robotic-assisted minimally invasive esophagectomy (RAMIE) was compared with conventional minimally invasive esophagectomy (cMIE) to evaluate postoperative outcomes [9]. Overall, the evidence showed that RAMIE achieved comparable results to cMIE in terms of complication rates and recovery in most cases [9]. However, in certain surgical approaches, such as the Ivor Lewis technique, robotic surgery was associated with improved clinical outcomes, particularly regarding lower postoperative morbidity [9]. These findings suggest that while RAMIE may not provide significant advantages over cMIE in all contexts, it can offer meaningful benefits in selected procedures that require enhanced precision and control [9].

Furthermore, a study included 95 morbidly obese patients who underwent hysterectomy for endometrial cancer [10]. Surgical methods were robotic-assisted in 36.8%, laparoscopic in 40%, and vaginal in 23.2% of cases [10]. Baseline patient characteristics and cancer staging were comparable across all groups [10]. The results showed no conversions to open surgery in the robotic group, whereas 5.3% of laparoscopic procedures required conversion due to complications [10]. Operative time was significantly longer in the robotic-assisted group, but hospital stay and postoperative complication rates did not differ significantly between the approaches [10]. Severe complications were infrequent, and no mortality occurred within 90 days postoperatively [10].

While we have focused on patient outcomes, it is equally important to consider surgeon-related outcomes, including the physical demands and ergonomic challenges associated with different surgical approaches. Several studies indicate differences in muscle stress patterns and postures between traditional laparoscopic surgery (TLS) and robotic-assisted surgery (RALS) [11]. Surface Electromyography (sEMG) analysis showed that TLS activates more of the arm and shoulder muscles, while RALS stresses the lower back, neck, and finger muscles [11]. Although RALS reduces upper-extremity fatigue, it creates stress in other areas due to the nature of sitting and hunching over the console [11]. Subjective measures, such as the Borg CR10, which is a physical exertion scale, have also shown that surgeons experience greater lower back fatigue when using RALS, which may be related to poor chair settings or insufficient awareness of optimal posture [11].

Posture assessment tools such as Rapid Entire Body Assessment (REBA) and Rapid Upper Limb Assessment (RULA) have been used to determine the appropriateness of postural adjustments during surgery and have shown that TLS results in unbalanced standing postures, while RALS can lead to trunk and neck flexion [11]. RULA is recommended for RALS assessments, and REBA for TLS assessments [11]. These findings underscore the importance of education and training in maintaining healthy posture and proper ergonomics to reduce chronic muscular stress in surgeons [11].

Taken together, these findings highlight the urgent need to integrate biomechanical principles training into surgical programs, especially with the increasing reliance on robotic surgical systems. Furthermore, the variation in stress across different body areas depending on the type of technology indicates that the solution lies not only in the technology itself, but also in its integration with user awareness and modifications to the surgical work environment. Therefore, improving surgical outcomes is not limited to the patient alone; it must also include reducing the physical burden on the surgeon to ensure sustained performance and minimize the risk of long-term musculoskeletal strain.

Based on findings from various studies, a balance in outcomes between robotic and laparoscopic surgery has been observed across surgical procedures. While robotic surgery offers many advantages, such as lower complication rates, faster recovery, shorter hospital stays, and reduced conversion to open surgery, it is associated with longer operative times and higher costs. Overall, robotic surgery is a promising option in some cases, particularly for achieving better long-term clinical outcomes, but laparoscopic surgery can be faster and more cost-effective in others. Future studies should continue to compare these techniques more comprehensively to determine where each technique excels, considering costs, complications, and patient outcomes.

## COMPLICATIONS

Complications are among the most important factors in assessing the safety and overall success of any surgical technique. Complications have a significant impact on patient recovery, hospital stay, healthcare costs, and long-term outcomes. With the rapid growth of robotic surgery across specialties, it has become increasingly important to determine whether robot-assisted surgery results in lower complication rates than traditional laparoscopic techniques. This section reviews the evidence comparing robotic-assisted surgery with laparoscopic surgery in terms of postoperative complications.

A recent meta-analysis of 51 studies comparing robotic gastrectomy with laparoscopic gastrectomy found that robotic gastrectomy was generally associated with lower postoperative complications [12]. The overall complication rate was lower in patients who underwent robotic surgery [12]. Regarding major complications, the study indicated that robotic surgery was associated with improved outcomes [12]. Patients in the robotic surgery group experienced fewer severe complications, such as those requiring surgical intervention [12]. Additionally, robotic surgery was associated with lower rates of specific complications, including anastomotic dehiscence, intra-abdominal infections, fluid accumulation, pneumonia, and pancreatic fistulas [12]. However, there were no significant differences between robotic surgery and laparoscopic surgery in terms of intra-abdominal bleeding risk [12].

In the context of pancreatic surgery, postoperative pancreatic fistula remains a major concern. Comparative studies report similar rates of postoperative pancreatic fistula between the two techniques, with overall incidences of 39.2% in the robotic group and 42.3% in the laparoscopic group, with no statistically significant difference [13]. Regarding reoperation rates, which reflect the need for additional surgical intervention due to complications, results were also comparable [13]. In multiple studies, the percentage of patients requiring re-exploration was slightly lower in the robotic surgery group (2.45%) compared to the laparoscopic surgery group (4.39%), but this difference did not reach statistical significance [13]. Conversion to open surgery was more frequent in the laparoscopic group (15.7%) compared to the robotic group (4.9%), although the supporting evidence remains limited [13]. Postoperative mortality rates were low in both groups. The pooled (weighted) mortality rate was 1.7% for laparoscopic procedures and 0.4% for robotic procedures [13]. Overall, the studies indicate comparable safety levels between both techniques regarding postoperative complications, with robotic surgery showing slight advantages in reducing reoperation rates and postoperative mortality.

A recent meta-analysis compared Robotic Intersphincteric Resection (R-ISR) with Laparoscopic Intersphincteric Resection (L-ISR) for lower rectal cancer; the results showed no significant difference in postoperative complications between the two techniques [14]. The analysis revealed no significant difference in the incidence of anastomotic leak between the two groups, nor was there a significant difference in the rates of postoperative intestinal obstruction [14]. Furthermore, the incidence of intra-abdominal abscesses did not differ significantly between the robotic and laparoscopic approaches [14]. However, robotic-assisted surgery was associated with lower rates of postoperative urinary tract complications than laparoscopic surgery [14]. This finding may reflect that the robotic platform provides better visualization and precision, which may help protect surrounding pelvic structures. Overall, the study concluded that both surgical techniques are generally safe, with robotic-assisted surgery demonstrating lower rates of postoperative urological complications. Furthermore, a systematic review and meta-analysis comparing robotic-assisted radical cystectomy with laparoscopic radical cystectomy found no significant differences in complication rates during surgery or in the early postoperative period (within 30 days) [15]. However, robotic-assisted surgery was associated with lower rates of long-term postoperative complications (within 90 days or more) [15]. These findings suggest that although both techniques demonstrate similar safety levels and short-term postoperative complications, robotic-assisted surgery may offer an advantage in reducing long-term postoperative complications.

In summary, while several studies suggest that robotic-assisted surgery may offer modest benefits in reducing certain postoperative complications, such as severe complications, urinary tract infections, and reoperation rate, as shown in the intersphincteric resection study for low rectal cancer, other evidence suggests similar safety profiles between robotic and laparoscopic approaches for various procedures.

The results highlight the importance of considering the surgical setting, procedure type, and surgeon experience when comparing complication risks of different surgical techniques. There is a need for more high-quality comparative studies to confirm the consistency of these advantages across broader patient populations and surgical specialties.

## RECOVERY

When comparing surgical approaches, patients’ recovery times for each technique must be considered to assess their effectiveness. A faster recovery time is often associated with reduced postoperative discomfort, shorter hospital stays, earlier return to normal functions, and improved patient satisfaction. With the rise in the use of robotic-assisted surgery, there is a growing interest in understanding whether this surgical approach can speed up recovery time after surgery compared to laparoscopic surgery. This section highlights evidence comparing robotic-assisted surgery and laparoscopic surgery with respect to recovery time, return to normal function, and hospital stay.

Results from studies comparing robotic and laparoscopic colorectal surgery showed that recovery time may be shorter with robotic surgery for some types of resection [16]. Specifically, the results showed that patients who underwent robotic left hemicolectomy, proctectomy, and rectosigmoid resection experienced shorter recovery time and earlier return of bowel functions than those who underwent laparoscopic surgery [16]. However, no differences in bowel recovery time were observed after right hemicolectomy [16]. Similarly, the hospital stay was shorter in the robotic group for the same three surgical procedures (left hemicolectomy, proctectomy, and rectosigmoid resection), while it remained comparable between the two groups for right hemicolectomy [16]. Although the results indicated that robotic-assisted surgery was superior in shortening recovery time and hospital stay, the outcomes for bladder cancer patients who underwent robotic and laparoscopic radical cystectomy yielded different findings [15]. A systematic review and meta-analysis compared robotic-assisted radical cystectomy with laparoscopic radical cystectomy in bladder cancer patients, analyzing data from 14 studies including 1,939 patients [15]. The analysis found no significant difference in hospital stay between robotic-assisted and laparoscopic surgery [15]. Also, there were no significant differences in short-term recovery measures, such as time to oral feeding and time to return to a normal diet [15]. These results suggest that both robotic-assisted radical cystectomy and laparoscopic radical cystectomy provide similar recovery experiences [15]. Several findings from non-randomized studies indicate that patients who underwent robotic-assisted radical prostatectomy recovered more quickly after surgery than those who underwent the laparoscopic approach [4]. Robotic-assisted radical prostatectomy was associated with a shorter catheterization time and hospital stay compared to laparoscopic surgery [4]. These findings suggest a faster initial recovery for patients who underwent robotic-assisted radical prostatectomy [4]. Furthermore, patients who underwent robotic-assisted radical prostatectomy experienced faster functional recovery, particularly in terms of urinary control and erectile function [4]. Furthermore, a study comparing robotic-assisted cholecystectomy with laparoscopic cholecystectomy found that hospital stay was shorter for patients who underwent robotic cholecystectomy [8]. These results may reflect a faster recovery and a shorter convalescence period after surgery in the robotic group [8]. In a study comparing robotic and laparoscopic gastrectomy for gastric cancer patients, postoperative outcomes were assessed based on length of hospital stay, time to first passing gas, and time to initiation of oral fluid or food intake to measure recovery [12]. The results showed shorter recovery time, shorter time to first passage of gas, and earlier initiation of oral fluid and food intake in the robotic surgery group compared with the laparoscopic group [12]. These results suggest that robotic surgery is generally associated with shorter recovery than laparoscopic surgery [12].

In procedures such as esophagectomy, robotic-assisted minimally invasive surgery has shown comparable recovery outcomes to conventional minimally invasive [9]. While some studies have found no significant differences in metrics such as hospital stay duration or ICU readmission rates, others have reported a trend favoring robotic surgery in achieving functional recovery milestones more quickly [9]. These include earlier mobilization, better pain control, and quicker return to oral intake, suggesting that the enhanced precision and stability offered by robotic platforms may support a smoother recovery process in select cases [9].

Overall, the studies showed that robotic-assisted surgery was associated with significant improvements in recovery time compared to laparoscopic surgery, including shorter hospital stays and faster return to normal function. These benefits contribute to improved health care quality and increased overall patient satisfaction. However, these benefits vary by surgical specialty, underscoring the need for further studies to evaluate the effects of robotic surgery across specialties.

## COST

In addition to patient outcomes, complication rates, and recovery time, comparing surgical costs between robotic surgery and laparoscopic surgery has become an important factor in surgical decision-making. Since surgical costs are an important factor in determining the effectiveness of treatment for both healthcare systems and patients, comparing the economic differences between robotic and laparoscopic approaches is essential. This section explores the differences between robotic-assisted surgery and laparoscopic surgery in terms of surgical costs, drawing on recent studies and cost-effectiveness analyses.

**Table 1:** Key comparative summary of robotic surgery vs. laparoscopic surgery

Aspect	Robotic surgery	Laparoscopic surgery
**Surgical precision**	High precision, especially in complex procedures.	Good precision, but limited dexterity in certain procedures.
**Complication rates**	Slightly lower in some procedures.	Generally low and comparable; may be higher in complex cases.
**Recovery time**	Often faster recovery and shorter hospital stay.	Comparable recovery in many cases; sometimes slightly longer.
**Conversion to open surgery**	Lower conversion rates.	Higher conversion risk.
**Operative time**	Usually longer.	Shorter operative time.
**Cost**	Higher due to equipment and maintenance.	More cost-effective and accessible.
**Availability**	Limited to advanced centers; required specialized training.	Widely available and commonly practiced.
**Best use cases**	Complex surgeries requiring high precision.	Routine or less complex surgeries.

**Figure 1:**
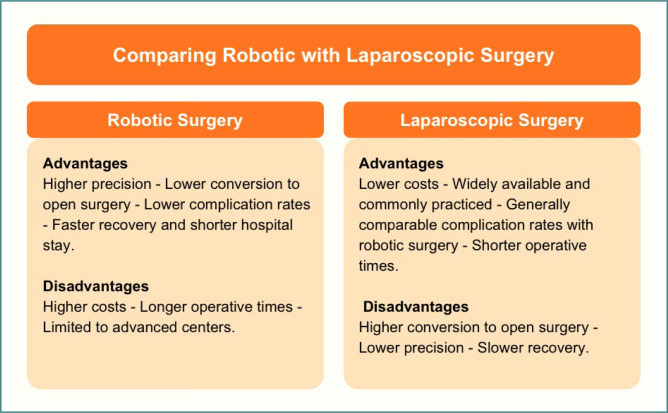
Comparison of robotic and laparoscopic surgery

In a meta-analysis comparing robotic-assisted gastrectomy and laparoscopic gastrectomy for gastric cancer patients, the results showed that robotic-assisted gastrectomy was associated with higher interventional costs [17]. The results revealed that robotic-assisted gastrectomy was 3,295.78 USD more expensive than laparoscopic gastrectomy, a statistically significant difference [17]. These results highlight the greater economic burden associated with robotic-assisted gastrectomy [17]. Additionally, a study found that the cost of laparoscopic surgery was approximately 16,000 USD, while the cost of robotic surgery was 18,300 USD, indicating a higher cost for robotic-assisted surgery [18]. Gastrectomy was the most expensive procedure for both robotic and laparoscopic techniques among all surgeries analyzed, with no significant difference in cost between the two approaches [18]. However, the costs of robotic-assisted procedures were consistently higher than those in laparoscopic surgery for major abdominal surgeries, with the largest gap estimated at 5,500 USD in gallbladder removal procedures [18]. After adjusting for multiple risk factors, robotic-assisted surgery was still associated with an average increase in hospitalization costs of 3,000 USD compared to laparoscopic surgery [18]. Moreover, a pooled analysis of five studies involving 326 patients showed that robotic liver resection was more expensive than laparoscopic liver resection [19]. Given the low heterogeneity across studies, a fixed-effects model showed a statistically significant increase in the overall cost of robotic liver resection compared with laparoscopic liver resection [19].

In summary, although robotic-assisted surgery offers technical advantages in complex cases that may outweigh those of laparoscopic surgery, it consistently incurs higher costs than laparoscopic surgery for a wide range of surgical procedures (Table 1). Even after adjusting for clinical and patient-related factors, the cost disparity remains significant. Since surgical costs are among the most important factors influencing decision-making and surgical planning, there is a need for more studies to determine whether robotic surgery is worth the high costs and how to reduce the costs associated with robotic surgery (Figure 1).

## DISCUSSION

This narrative review aimed to compare robotic-assisted surgery and laparoscopic surgery in terms of patient outcomes, postoperative complications, recovery time, and cost across different surgical specialties and procedures. Analysis of studies comparing robotic and laparoscopic surgery has shown that both techniques offer benefits and advantages as minimally invasive approaches, though each has its own limitations.

One key finding in comparing surgical approaches across disciplines is the overall positive patient outcomes associated with robot-assisted surgery. While robotic-assisted surgery was associated with longer operative times, it demonstrated lower conversion rates to open surgery and lower postoperative complication rates than laparoscopic surgery [2,7]. This may explain the potential of robotic surgery to enhance precision and intraoperative control, especially in complex anatomical areas. However, it is important to note that overall complication rates between robotic-assisted and laparoscopic surgery have shown similar results in other surgical procedures, with slight advantages in favor of robotic-assisted surgery in specific contexts [10,13].

In terms of recovery, robotic surgery has shown a trend toward shorter hospital stays and faster functional recovery than laparoscopic surgery for several surgical procedures, including colorectal resection, cholecystectomy, and gastrectomy [2,7,8,12]. This may be attributed to the lower likelihood of tissue damage and the high precision of robotic systems. Additionally, robotic-assisted radical prostatectomy has been associated with faster recovery of normal functions such as bladder control and erectile function [4], which may improve quality of life in the short term. However, various studies comparing robotic-assisted radical cystectomy and laparoscopic radical cystectomy have shown no significant differences in the recovery time between robotic and laparoscopic surgery [15].

The high cost associated with robotic-assisted surgery is a significant factor hindering its widespread adoption. The studies mentioned above have shown that robotic surgery is associated with higher costs than laparoscopic surgery across various surgical procedures, even after adjusting for patient clinical factors [17, 18, 19]. The high cost of robotic equipment, maintenance, and longer operative times are the primary factors driving the high costs of robotic surgery, which in turn contribute to increased hospital costs. Despite the clinical benefits that robotic surgery provides, its high costs raise important questions about its cost-effectiveness, especially in settings with limited resources or in surgical procedures where robotic surgery yields results similar to laparoscopic surgery but at a higher cost.

There are additional factors to consider when interpreting the results, such as differences in study design, surgeon experience, and healthcare environments across the published studies. Also, variations in surgical experience and the learning curve can affect surgical outcomes, making it difficult to attribute results solely to the technology. These limitations highlight the need for well-designed, prospective, multicenter randomized controlled trials to provide more definitive evidence on the effectiveness of robotic-assisted and laparoscopic surgery.

Regarding the potential use of artificial intelligence (AI) to evaluate which of the two surgical techniques is optimal for different surgical procedures, several recent studies have demonstrated a growing trend toward integrating AI technologies into the preoperative planning phase of robotic surgery to improve decision-making and predict surgical outcomes [20]. These studies have employed various models, including logistic regression, machine learning algorithms (e.g., XGBoost), and neural networks, to analyze clinical data and radiological images, such as computed tomography (CT) and magnetic resonance imaging (MRI) [20]. These models have improved the ability to predict surgical difficulty, the likelihood of conversion to open surgery, and postoperative outcomes such as urinary control and erectile function, reflecting the increasing value of AI in improving the quality of surgical care [20].

Overall, this trend reflects a significant shift in modern surgical practice, particularly in terms of enhancing predictive accuracy and improving patient outcomes. The importance of these predictive models lies in their ability to support surgeons in decision-making based on multidimensional data, aligning with the objectives of this research to explore the impact of integrating AI technologies on improving surgical efficiency and reducing potential complications. The diversity of tools used in previous studies demonstrates the flexibility and adaptability of AI to different surgical types and available data, opening promising prospects for future research and development in this field.

Based on the findings of this study, procedures should be clearly identified according to their relative outcomes: those that demonstrate improved results when performed using robotic surgery, and those for which robotic surgery provides no significant advantage but is instead associated with higher costs and longer operative times. Despite benefits in precision, lower complication rates, and faster recovery in procedures such as radical prostatectomy (urology), benign hysterectomy (gynecology), and left-sided colorectal resection (colorectal surgery), these advantages have not been consistently observed across all surgical specialties. Specifically, procedures such as right hemicolectomy, pancreatic surgery, and radical cystectomy did not demonstrate statistically significant differences in outcomes when compared to laparoscopic surgery [13, 15, 16]. Furthermore, these procedures were associated with significantly higher costs and longer operating times when performed robotically.

Therefore, the integration of robotic surgery should be carefully considered on a case-by-case basis. For procedures where robotic platforms do not demonstrate clear superiority—such as right hemicolectomy, pancreatectomy, or radical cystectomy —laparoscopic surgery remains a more efficient and cost-effective option. This aligns with previous findings that robotic access may not be advisable for some surgical procedures unless anatomical or patient-specific factors justify its use. These observations highlight the need for procedure-specific guidelines for the use of robotic platforms, balancing clinical benefits, potential risks, and resource allocation.

Furthermore, while robotic-assisted surgery offers significant clinical advantages in many cases and procedures, especially in those that require high precision, it is unlikely to completely replace laparoscopic surgery in the near future. The high cost and limited accessibility of robotic platforms remain significant challenges, particularly in low-resource settings. A more balanced, case-specific approach may be necessary, selecting the surgical approach based on the patient’s condition, institutional capabilities, and the complexity of the procedure. Future research should also explore procedures where robotic surgery consistently outperforms laparoscopic techniques, and vice versa. Furthermore, future studies should focus on developing cost-reduction strategies and the long-term impact of robotic-assisted surgery on patient-centered outcomes, such as quality of life, return to normal activity, and functional recovery.

## CONCLUSION

In conclusion, this narrative review highlights that both robotic-assisted surgery and laparoscopic surgery offer several benefits and advantages as minimally invasive surgical techniques. Robotic-assisted surgery has advantages such as increased precision, reduced need for conversion to open surgery, fewer postoperative complications, faster recovery, and shorter hospital stays. However, laparoscopic surgery offers advantages, including lower surgical costs and greater accessibility, especially in settings with limited resources. While robotic surgery demonstrates improved outcomes in complex procedures, its widespread adoption remains challenging due to its high costs and limited availability. Therefore, the choice of robotic or laparoscopic surgery for a given procedure should be based on patient-specific factors, surgical complexity, and the resources available at the medical institution. High-quality, prospective, randomized, and multicenter studies are needed to further evaluate long-term clinical outcomes, cost-effectiveness, and patient-related benefits across different surgical specialties and procedures.

## Data Availability

The datasets used and analysed during the current study are available from the corresponding author upon reasonable request.

## Personal thanks

We would like to thank all the participants in this study. We would also like to express our gratitude to the Deanship of Scientific Research at King Faisal University for their support.
